# Nanotechnology in agriculture: a review of genotoxic studies of nanopesticides in animal cells

**DOI:** 10.1007/s11356-023-26848-y

**Published:** 2023-04-28

**Authors:** Cynthia Paz-Trejo, Ana Rosa Flores-Márquez, Sandra Gómez-Arroyo

**Affiliations:** Laboratorio de Genotoxicología Ambiental, Instituto de Ciencias de la Atmósfera y Cambio Climático, Universidad Nacional Autónoma de México, Ciudad Universitaria, México

**Keywords:** Nano pesticides, Pesticides, Genotoxicity, Nanotechnology, Risk evaluation

## Abstract

Agriculture has been and still is one of the most influential primary operations in economic history worldwide. Its social, cultural, and political impact allows the progression and survival of humanity. Sustaining the supply of primary resources is crucial for the future. Therefore, the development of new technologies applied to agrochemicals is growing to obtain better food quality faster. Recently, nanotechnology has gained strength in this field in the last decade, mainly because of the presumed benefits that will carry with it compared with the current commercial presentations, like the decrease of risk in non-target organisms. The harm of pesticides is commonly associated with unwanted effects on human health, some with long-term genotoxic effects. Therefore, it would be relevant to set the existence of a risk or a benefit of the nanopesticides from a genotoxic point of view, comparing against those without this technology. Although some studies are concerned with its genotoxicity in live aquatic organisms, few focus on human in vitro models. Several studies conclude that some of them can induce oxidative stress, leading to DNA damage or cell death. However, there is still much to investigate to establish an accurate and complete assessment. In this review, we aim to give an overview of the genotoxic effect caused by nanopesticides in animal cells and a guide to the evolution of this topic, offering a base and critical review to facilitate future research.

## Introduction

The application of nanotechnology in agrochemicals has been rising since the beginning of the XXI century, intending to improve crop production and make them more sustainable.

Due to the fast growth, the Food and Agriculture Organization (FAO) requested a discussion around three main concerns: the use of nanotechnology in food, the potential risk of its use in human health, and the social aspects of the acceptance between the communities (FAO 2002).

However, there are still differences in the regulation of nanotechnology around the world concerning definition, labeling, nanospecific notification, and guidance on toxicity or risk assessment (Kihara 2021). Even when Australia, Canada, The European Union, and the United States of America (USA) have regulations on the definition of industrial chemicals (where there are supposed to be agrochemicals), there still need to be specifications on the labels of the products. This practical gap has led to the innovation and growth around the use of this technology in pesticides, where Asia is the actual leader in academic production, and the ones with the most patents of nanomaterials directed to agriculture are the USA and Germany (Kah et al. 2013).

Despite studies on the efficacy of these products in the field, most of the nanopesticides still need to be included in the nanoscale definition (1–100 nm). However, the term is in use because the products have entities within the range of 100–1000 nm, are also designated with the prefix “nano,” or have novelty properties associated with the size (which is relevant for the definition of nanomaterials) (Kah and Hofman 2014). These new nanometric presentations may imply a new risk from previous chemicals with no harmful effects associated with any system.

Due to the novel characteristic of nanotechnology, studies made with metallic nanoparticles like silica, aluminum, or titanium oxide used in construction materials and others, have supported the easy diffusion, resistance, dimensionality, and entry to biological systems (Kumar and Dhawan 2013).

Also, the knockdown of genes associated with the cell cycle was found in epithelial lung cells exposed to copper nanoparticles (Hanagata et al. 2011). The nanopresentation might have different behaviors than the bulk presentation, so predicting their toxicokinetic and toxicology behavior will be necessary; however, they still need more genotoxic research to prevent advance in this knowledge. The revision of Oberdörster et al. (2006) debates the potential risk of the size reduction in commercial products concluding that the reduced size might allow easy access throughout epithelial and endothelial cells. They can also imitate molecules associated with catalysis reactions, mainly because their atomic and molecular surface plays a relevant role in increasing their activity in biological systems, even at the genetic level.

Experiments in the production of nanoparticles have shown that absorption and adsorption increase in these particles. Depending on the uptake into the system, they can persist in blood and have a systemic distribution.

The elimination of non-biodegradable nanoparticles is slow and inefficient and could lead to oxidative stress inflammation (Magdolenova et al. 2012). It has also been reported that lipid peroxidation in the brain and erythrocytes (Fetoui et al. 2015). Of all this evidence, they may induce genetic damage. Yet, we are still determining the amount of information or research about the genotoxic damage of nanopesticides in human cells.

In this review, we aim to give an overview of the genotoxic effect caused by nanopesticides in animal cells and a guide to the evolution of this topic, offering a base and critical review to facilitate future research.

### The impact of agriculture on the world

Agriculture is a primary operation worldwide, followed by cattle raising, fishing, mining, and forest exploitation. Its impact on human life is crucial; it provides food and raw materials for populations and forms the axis of society’s economy.

It has been so crucial that in 1999 the grass and crop fields extended over the 37% of arable land globally, increasing by 10–15% nowadays, estimated that by 30 years from now, developing countries would need 120 million extra hectares for agriculture due to the population growth (FAO 2015).

Unfortunately, half of the suitable land for agriculture usable in the future is in just seven tropical countries in Latin America and Africa (FAO 2015). This is because the products needed to sustain a future population require intense agricultural cycles in short periods. In addition, these areas have specific physical, edaphological, and environmental characteristics that make them ideal for sowing and are relevant because every agricultural development relies on the area’s natural resources and is restricted to these properties.

There are few countries with the territory, workforce, and environmental capacity suitable for quickly producing a large amount of food. Some are China, India, Brazil, Indonesia, and Latin America (FAO 2018).

However, there are some leading countries in production not by territory but by agricultural improvements, like the USA, Russia, and Ukraine.

This improvement has allowed places with unfavorable climatic altitudes can compete with those countries with optimal environmental conditions to produce food that usually could not be obtained so easily, for example, the sowing of corn between Brazil and the USA as leaders in production (FAO 2018). This advantage has, undoubtedly, been brought about by agrochemicals like fertilizers and pesticides.

### The relevance of the use of pesticides and their evolution

The need to protect crops against pests was born out of the fear of loss. And despite ancient writing of the use of sulfur as a fungicide in 1000 A.C. by Homer (Porcuna 2010), it was until the twenty centuries with the Second World War that the exploration and research around synthetic organic products started, given by the first time the possibility of developing pesticides (Sánchez Raya 2002).

In the 40 s decade, many substances used as pesticides were created as chemical weapons for the Second World War. Their properties as pesticides were explored, like DDT in 1942; Paul German Müller discovered its insecticide properties for the Sweis Geigy Company. However, it was already implemented as a weapon in 1874 (Sefy 2020). These products’ advantages were numerous, so in 1948 Müller won the Nobel Prize for saving millions of lives by applying the insecticide against organisms that caused illness and protecting dozens of crop fields.

The first generation of pesticides was born, whose principal representatives were 2,4-dichlorophenoxyacetic acid (2,4-D) and dichlorodiphenyltrichloroethane (DTT). Their incursion on the market was economically advantageous worldwide, producing the second generation of pesticides between 1945 and 1955, where organophosphates, carbamates, and ureas were the representatives (EPA 2018).

The development and discovery of these substances have been steadily increasing, leading to the exploration of new product presentations to make them more efficient and safer for human beings.

### The impact of pesticides on the environment and health

The first generation of pesticides focused on rediscovering properties from war chemical weapons to pesticides, reusing and synthesizing based on existing products, substances efficient to destroy plagues but harmful to human health and the environment.

Nevertheless, at the beginning of the 70 s, the population started to realize the environmental repercussion of pesticides. In 1962 Rachel Carson, an American biologist, brought out “Silent Spring,” where she warns about the toxicological and harmful effects of pesticides on the environment; this writing was consolidated as the first disclosure of evidence of the toxicology of pesticides and allowed the dialog and debate about the benefits and damage around them (Carson 2016). It had such an impact that in 1972, DTT was banned from the USA, starting the development of a new generation of less hazardous pesticides (pyrethroids and sulphonylurea)—leading to a new path of continued innovation through history with one purpose only: to create an efficient product with a specific target.

One of the existing problems has been developing pesticides with a specific target since now it only relies on the dosage and time of application. This point is relevant and has been generating controversy through the years since it is the first way of exposition for the people in contact with them.

In the 80s, the relevance of pesticides was evident, yet, it led to a debate between the toxicological and ethical studies of people in contact with them. Hence, the Food and Agriculture Organization (FAO), to control and regularize their implementation and production, elaborated The International Code of Conduct for the Sustainable Use and Management for Fertilizers with an actualization in 2002 (FAO 2002; Sánchez Raya 2002).

Finally, pesticides were considered in all subsequent global agreements like the Montreal Protocol, Rotterdam, and the Stockholm Convention. Several associations were made for its control, classification, regularization, and toxicological evaluation in different parts of the world, such as OMS, FAO, PAN (Pesticide Action Network International) (PAN 2015), EPA (U.S. Environmental Protection Agency) (EPA 2015), IARC (International Agency for Research on Cancer) (IARC 2015), and in México (mainly), La Comisión Federal para la protección contra Riesgos Sanitarios (COFEPRIS 2022).

### Innovation and nanotechnology

The historical, social, and political impact of pesticides in agriculture has been broad and inevitable. Promoting its growth and development, and more importantly, its innovation and application of new technologies with one goal: to develop a product efficient in the field and little or non-harmful to human health and the environment.

Nowadays, 42% of the population worldwide works in agriculture (FAO 2019), and it is presumed that economic increase and poverty reduction are linked to agricultural growth. So, any technological implementation in agrochemicals will allow improvement in this field.

The first nanotechnology industry was born at the beginning of the 90 s, bringing novelty properties used in different public and industrial sectors. Its impact was so vast that in 2004, the Royal Academy of Engineering of Spain released a statement insisting on the restriction of its use, mainly because there was little knowledge about its safety in the application (ETC group 2004).

The big problem with this new technology relies mainly on the need for generalization around its definition worldwide. Several definitions exist for its implementation or physical characteristics, such as nanomaterial, nanoscale, nanoparticles, and nanoobject. One of the most accepted is the definition of “any material between 1 and 100 nm” defined by the European Union and other associations (European Commission 2015; JRC-IPTS 2014; WHO 2009); it still is not always applied. This gap around the standard definition has led to this technology’s limitless use and production worldwide.

Some of the newness advantages of the nanoparticles are more stability against their bulk presentation and resistance to drastic temperature changes (Wang et al. 2007). In addition, it increases aggregation capacity, photoemission, electric conductivity, and calorific and catalytic activity (Liu 2006). These characteristics have strengthened the implementation of nanomaterials in the construction and medicine industries (European Commission 2015) as well as in the food business, where the main goal is to improve its nutritional quality and where countries like Germany, the USA, China, and Israel are the primary producers (Woodrow Wilson International Center for Scholars 2009).

### Nanopesticides and the implications on human health

Early in 2010, nanotechnology started to be officially implemented in agrochemicals, using two definitions mainly: on the one hand, it constitutes something “novel” obtained through a synthesis of active ingredients with an optimal size between 100 and 400 nm, and on the other, consider a type of encapsulation that releases the active ingredient contained in it in a long way, as well as of the mechanism of action of the total formula as emulsions and solutions (Kah 2015; Kah and Hofmann 2014). In 2014, this growth accelerated exponentially, reaching hundreds of articles around the prefix “nano” followed by the word pesticide or fertilizer in development (Kah 2015).

The supposed benefits that will bring with it this new presentation are as follows: being cheaper and more “intelligent” than its current presentations, meaning increasing its efficiency, decreasing risk in no-target organisms and resistance development in target ones, more physically stable since it will prevent phases separation when are storage and apply, and its release can be directed in time, so the number of applications could be reduced (Kah and Hofmann 2014).

Since its a relatively new topic, research on its toxicity and health implications is scarce because most of the attention focuses on efficiency in the field and the environmental repercussions (Kookana et al. 2014; Nishisaka et al. 2014), so the genotoxic consequences, specifically in humans, have not been fully valued.

In 2003, Vyvyan Howard, editor and foundress of the “Journal of Nanotoxicology,” did a scientific review for the ETC (Grupo de Acción Sobre Erosión, Tecnología y Concentración) around the dangers of nanopesticides. She found that the toxicity is more due to its size and function because they can enter the body faster, crossing barriers such as the skin, placenta, blood, and brain (ETC group 2004).

It has been observed that pesticides can induce inherent toxicity, and through the years, many of them have been banned because of the genetic consequences that they can induce. However, nowadays, it is still controverting because there is no general regulation of them; the development of nanopesticides and their distribution on the market may be a risk to health even greater and is a concern because there are already more than 300 patents for nanopesticides, and some are already in use (Kah and Hofmann 2014).

## Methodology

The central purpose of our systematic literature review is to provide a critical, analytical account of the literature available from 2010 to 2022 concerning the main genotoxicity and some toxicity events (like oxidative stress) of nanopesticides in animal cells.

A thorough revision was made using PubMed, Scopus, Microsoft Academic, and Base for the results section to achieve this. Since the topic is somewhat recent, and one of the main goals is to analyze the type of scientific information available on the subject, only a few exceptions were made in the search.

The recent study used the search keywords “Genotoxicity,” “Genotoxic,” “DNA damage,” “Toxicity,” and “Risk assessment,” followed or preceded by “Nano pesticide,” “Nano agrochemical,” or “Nanoparticle”; and were chosen only the papers that contain information about the main molecules use as pesticides that can be found in a nanopresentation: titanium dioxide (TiO_2_), zinc oxide (ZnO), copper (Cu), pyrethrin, valinomycin, and lambda-cyhalothrin. Only original research was considered in Table 1 for a profound analysis, both in English and Spanish, and the accessibility to the article should be through the researchers’ university or professional email; also, articles concerning toxicity and genotoxicity of nanopesticides or nanoparticles used as pesticides in vivo or in vitro studies were considered. The articles without statistical analysis were discarded.

**Table 1:** Effect of nanopesticides in different study models

Authors	Year	Product	Main evaluation tests used to evaluate damage	Model	Did it induce damage or molecular response (yes/no)	Type of response
Cho et al	2010	Nanoparticles: TiO_2_ Cu	Histology Immunohistochemistry	In vivo*:* The lung of Wistar rats	Yes, for both metals	Inflammation
Liu et al	2010	Nanoparticles: TiO_2_	Flow cytometry	In vitro*:* Brain neurons (PC12 cells)	Yes	Increase the amount of oxidative stress by reactive oxygen species, and apoptosis
Rossi et al	2010	Nanoparticles: TiO_2_	PCR amplification Enzyme-linked immunosorbent assay	In vivo: *BALB/c mice* In vitro*:* Human macrophages Human Fibroblasts (MRC-9)	No	Inflammation
Shi et al	2010	Nanoparticles: TiO_2_	HPLCEC to determine 8-OhdG Micronucleus tests Comet assay	In vitro*:* Human embryo L-02 Hepatocytes	No	It did not increase DNA damage but, it did increase the amount of oxidative stress by reactive oxygen species and the amount of 8-OhdG
Shin et al	2010	Nanoparticles: TiO_2_	Real-time PCR Western blot hybridization Immunofluorescence staining Nuclear extract preparation and electrophoretic mobility shift assay (EMSA)	In vivo*:* Normal and septic brains of male C57BL/6 mice	Normal brains: no Septic: yes	Inflammation
Hackenberg et al	2011	Nanoparticles: ZnO	Comet assay ELISA test	In vitro*:* Primary human nasal mucosa cells	Yes	Genotoxic damage and inflammatory response
Magdolenova et al	2012	Nanoparticles: TiO_2_	Comet assay Comet assay modification to determine 8-OhdG	TK6 human lymphoblast cells EUE human embryonic epithelial cells Cos-1 monkey kidney fibroblasts	Yes	DNA damage and only DNA oxidation lesions were detected in Cos-1 and TK6 cells
Li et al	2012	Nanoparticles: ZnO	Micronucleus test	In vivo*:* Male ICR mice In vitro*:* Human umbilical cords Human umbilical vein endothelial cells (HUVECs)	Yes for both	Genotoxic and cytotoxic damage induced by oxidative stress
Perreault et al	2012	Nanoparticles: CuO	DNA fragmentation assay in agarose gel electrophoresis DNA methylation Micornucleus test	In vitro*:* Mouse neuroblastoma cell line Neuro-2A	Yes	Genotoxic damage
Rotoli et al	2012	Nanoparticles: TiO_2_ CuO	Fluorometric assay using 5-(and-6)-chloromethyl-20,70 -dichlorodihydrofluorescein diacetate	In vitro*:* Calu-3 epithelial cells Raw264.7 macrophages	Yes for both	Increase the amount of oxidative stress by reactive oxygen species
Di Bucchianico et al	2013	Nanoparticles: CuO	Micronucleus test Comet assay	In vitro*:* Murine macrophages RAW 264.7 Peripheral whole blood from healthy volunteers	Yes	Genotoxic damage
Fetoui et al	2015	Lambda-cyhalothrin	Micronucleus test	In vivo*:* Adult male Wistar rat	Yes	Genotoxic damage
Meredith et al	2015	Nanosized capsule of lambda-cyhalothrin	Embryonic zebrafish assay	In vivo*:* Danio rerio	Yes	Teratogenic problems
Ruiz et al	2015	Nanoparticles: CuO	Micronucleus test Real-time PCR Histology	In vivo*:* Mussels *Mytilus galloprovincialis*	Yes	Genotoxic damage
Uzar et al	2015	Nanoparticles: ZnO	Comet assay	In vitro*:* Rat kidney epithelial cells (NRK-52E)	Yes	Genotoxic damage
Sundaramoorthy et al	2016	Nanopermethrin	Micronucleus test	In vitro*:* Human peripheral erythrocyte/lymphocyte	Yes	Genotoxic damage
Di Bucchianico et al	2017	Nanoparticles: TiO_2_	Comet assay Micronucleus test	In vitro*:* BEAS-2B cells	Yes	Genotoxic damage
Patel et al	2017	Nanoparticles: TiO_2_	Comet assay Chromosome aberration assay (CA assay)	In vitro*:* Human peripheral blood cultures	Yes	Genotoxic damage
Carmona et al	2018	Nanoparticles: CuO	Wing-spot tests	In vivo*:* *Drosophila melanogaster*	No	Genotoxic damage
Oliveira et al	2019	Nanoparticles loaded with pyrethrum extract	Comet assay	In vivo*:* Bullfrog tadpoles *(Lithobates catesbeianus)*	Yes	Genotoxic damage
Brandao et al	2020	Nanoparticles: TiO_2_	Micronucleus test	In vitro*:* Lung cells (A549) Liver cells (HepG2) Glial cells (A172) Neurons (SH-SY5Y)	No	Genotoxic damage
Famhy et al	2020	Nanoparticles: CuO	Comet assay	In vitro*:* Human lung normal cell lines (WI-38) Human lung carcinoma cell (A549)	Yes	Genotoxic damage
Demir et al	2022	Nanopermethrin Nanovalidamycin Nanoparticles of CuO	Comet assay Real-time PCR	In vivo*:* Drosophila melanogaster	Yes	Genotoxic damage
Paz-Trejo et al	2022	Nano capsule of lambda-cyhalothrin	Comet assay Micronucleus test	In vitro*:* Human peripheral blood cultures	Yes	Genotoxic damage

A total of 124 articles were analyzed; only 25 were presented in Table 1 while another few were used to support definitions and data corroboration.

### Genotoxicity of nanopesticides

One problem with the current toxicological classification of pesticides is that most studies look for immediate or acute toxicity, dropping aside long-term or chronic damage, including genotoxicity (Bolognesi 2003). This last point is crucial because they can induce teratogenic problems, reproductive alterations, neurodegenerative disorders (like Parkinson’s and Alzheimer’s), and cancer (Costellos et al. 2009; Dosonmu et al. 2007).

The genotoxic study expands the search for diseases, long-term consequences, and even the indirect effect at the cellular level because genetic damage can be assessed directly or indirectly.

Since this technology is relatively new in production and implementation, toxicological research focuses on acute effects, environmental impact, and aquatic repercussions. Some examples were the furenos nanoparticles tested in Daphnia, which increased mortality and decreased fertility (Oberdörster et al. 2006). In addition, using copper nanoparticles proved the reduction in the action of Na/K channels in Zebrafish (Griffitt et al. 2009). Finally, the titanium dioxide (TiO_2_) nanoparticles decreased the activity of the glutathione enzyme in rainbow trout, being also toxic for the phytoplankton in combination with sunlight (Federici et al. 2007; Miller et al. 2012).

### The genotoxicological study is just beginning to gain strength

Two possibilities are currently exploring nanoparticles employed for non-agricultural purposes (now used as pesticides) and the pre-existent pesticides reformulated with a nanoparticle presentation or in nanocarriers.

Some of the most used compounds within the first category are titanium dioxide (TiO_2_), zinc oxide (ZnO), and copper (Cu), to name a few, while the most common ones in a new presentation are valinomycin, pyrethroids, and some new generations like lambda-cyhalothrin.

Table 1 shows the summarized information from the main original research concerning the analysis of the compounds mentioned above.

### Metallic nanopesticides

TiO_2_ is an insoluble compound, stable at high temperatures, cataloged with low toxicity, and inert at a physiological level (Iavicoli et al. 2011). However, in 2006, IARC (Shi et al. 2013) classified it as possibly carcinogenic in humans (Group 2B) because it has been observed to induce different toxicological effects in several organs and tissues.

In the nervous system, it also can induce reactive oxygen species as well as inflammation and apoptosis in mice (Liu et al. 2010; Shin et al. 2010); the internalization of the particles in the cell facilitates all of this. However, Rossi et al. (2010) mentioned that the evaluation of health risks cannot be solely based on pure nanoparticles; it is essential to consider modifying the nanoparticle’s characteristics.

Despite being TiO_2_, the most popular nanoparticle manufacturer worldwide, its exploration as a fungicide was recently explored (Sar and Unal 2017; Siddhartha et al. 2016). The most common presentation used is the nanoparticles between 10 and 80 nm; its effectivity is based on the disruption of the metabolism of the fungus generating oxidative stress damage (Shi et al. 2010; Shi et al. 2013).

These genotoxicity studies made with nanoparticles in cell lines have shown that the same effect proven in pathogens might reproduce in non-target organisms.

In lung cells (BEAS-2B), the effect was evaluated by micronucleus test and comet assay analyzed by flow cytometry and oxidation of bases with the enzyme Formamido Pyrimidine DNA Glycosylase (FPG) at 1–30 mg/mL for 3, 24, and 48 h. It was found that it induced damage in low concentrations in short times, mainly at 1 mg/mL, through the formation of micronuclei and a slight increase in DNA damage, evidenced by the test comments and activation of the FPG enzyme (Di Bucchianico et al. 2017). Also, it was evaluated in different cell lines like lung epithelium (A549), liver (HepG2), glia (A172), and neural (SH-SY5Y), through the comet assay and the micronucleus test being the first assay the one that evidence genetic damage. However, if cellular recovery is allowed without exposure to nanoparticles, the effect is decreased or disappears. Likewise, several concentrations and times were tested for each line. It was concluded that the level of induced damage depended on the cell type, the exposure time, and the concentrations, all this coupled with the physical and chemical characteristics that allowed the particle to interact with the different cell types, but most of the induced damage was repairable (Brandao et al. 2020). This information is relevant because one of the first and most important aspects to consider when evaluating nanoparticles is their physics and chemical properties since each behaves differently in the cells. Therefore, its evaluation and interpretation must consider; also, according to Cho et al. (2010), some molecular responses, like inflammation, presented differently in vitro tests than in vivo*,* so it is crucial to avoid making accelerated conclusions regarding the toxicology of nanomaterials.

The use of TiO_2_ as a pesticide is still in doubt since there is controversy around its harmful effects on human health; there is evidence of direct damage (chromosomic aberrations, mutation assays in mammals, sister chromatid exchange, among others), indirect induced by mechanisms or other elements aside from the DNA (reactive oxygen species, proteins photocatalysis, or harmful molecules for the DNA, among others) (Magdolenova et al. 2012; Patel et al. 2017), as well as negative results (Rossi et al. 2010). However, this should be addressed differently since most of the damage induced seems to be reparable.

Another joint compound used as a pesticide (fungicide) is ZnO. It is cataloged as “not particularly harmful” by the U.S. Food and Drug Administration (Sun et al. 2018). Its mode of action is based on the induction of oxidative stress and metabolic irruption, also inducing mycelium death (Barad et al. 2017; Tiwari et al. 2017).

One of the few existing genotoxic studies evaluated the exposition of ZnO nanoparticles in a kidney cell line (NRK-52E) by comet assay at 12.5–50 mg/mL by 24 h; the findings showed an increase in tail intensity confirming damage (Uzar et al. 2015); it was also evaluated its effect when was administrate orally in mice, where was proved that compare with its micrometric form; the nanoparticles were absorbed and distributed by the tissues without being metabolized, that is, they induce damage directly by oxidative stress, but no genotoxic damage was found. However, by administering intraperitoneal, the particles can remain in serum for a long time, and blood tests proved a genotoxic damage increase (Li et al. 2012). In vitro tests of nasal mucosa cells analyzed by comet assay, genetic damage was detected at 10–50 mg/mL, as well as an increase in the secretion of pro-inflammatory cytokines and the activation of superoxide dismutase (Hackenberg et al. 2011).

One point to consider when analyzing nanoparticles is the importance of using more than one genotoxicity study because, depending on the nature of the particle, it might require different methods to avoid false positives or false negatives, as well as modification of the techniques to evidence damage.

Likewise, the cupper is the third rising element used as a pesticide. This element has been used for years (mainly) as a fungicide, as part of the Bordeaux mix (CuSO_4_ and CaOH_2_), which was the first official combination used as a pesticide proved to stop snails and some algae (Vázquez-Blanco et al. 2020). There have been many explanations around the action of copper in different organic presentations, some being the capacity of the spores to accumulate it in small quantities until it reaches a toxic threshold, the release of copper when they touch the water from the organism, causing denaturing of the proteins and enzymes vital for the proper functioning of the body, among others (Porterfield 2018).

Copper has many advantages, such as easy storage and acquisition, low cost, and effectiveness. However, its prolonged use or large quantities can cause accumulation in food and the environment, for which it had to be modified to a more controlled presentation. Nowadays, pesticides are designed with copper, allowing a more efficient effect in crop fields. However, one of the biggest toxicological concerns around these nanoparticles is their effect on humans since the nanopresentation has different electric and biodynamic properties than their bulk presentation.

Studies made in the wings of *Drosophila melanogaster* through the “wing-spot” assay, comparing the effect of the nanoparticles versus microparticles, showed that none of them induced somatic mutations (Carmona et al. 2018). In some other aquatic organisms like the mussels, the genotoxic effect of the nanoparticles of copper versus its micropresentation by the micronucleus test and qRT-PCR of some genes (p53, Ras among others associated with cancer), as well as the oxidative stress through antioxidant enzymes (superoxide dismutase and catalase) was evaluated (Rotoli et al. 2012); results showed that nanoparticles increased the activity of the enzymes evidencing an elevated content of reactive oxygen species, inducing micronucleus. Still, they disappear after a recovery period, and the genes associated with cancer did not present significant deregulation (Ruiz et al. 2015).

Recent studies made in regular lung cell lines (WI-38) and tumorigenic (A549) show the induction of reactive oxygen species (ROS) and genotoxic damage through the increase of the tail length and percentage of tail DNA by the comet assay in both lines and conclude that the entrance to the cell it’s faster at lower concentrations (Fahmy et al. 2020).

Other studies made in neuroblastoma cells (N2A) observed the induction of micronucleus (Perreault et al. 2012); similar results were found in rodent macrophages and peripheral blood of donors exposed to different sizes of nanoparticles (Di Bucchianico et al. 2013).

Overall, these metallic particles (TiO_2_, ZnO, and Cu) are now used as pesticides in the industry. However, its application as a pesticide still leaves many questions ahead since its dynamics in the crop fields, health, and its new nanotechnology properties are unknown.

Most of the studies made around these nanoparticles have focused on aquatic organisms and cell lines associated with respiratory tracts, where it was observed, interestingly, that the majority induced genotoxicity is by oxidative stress (the most investigated mechanism by which the nanoparticles induce its effect) (Ye et al. 2010); although due to its size and easy access to the cell, they may induce direct damage as well since the particles can cross the epithelial barrier and cell membrane (Singh et al. 2009). It is also important to recognize that most of the genotoxic damage analyzed was made by cytogenetic assays like comet assay and micronucleus test, which can demonstrate damage of single and double-strand breaks.

### Active ingredients with nanotechnology implementation

Active ingredients synthesized as pesticides are innovating continuously to burst-specific cellular and systemic functions, intending to improve efficacy and diminish the unwanted effects on the environment and human health. Many of these products have diverse chemistry natures and are presented in different states (solids, liquids, gels, etc.) However, the nanotechnology application mainly looks to increase the potency and efficacy of active ingredients, little harmful, so that they can enter easily into the target organism and release it on the wanted area, but not do it in non-target organisms. Nevertheless, it is still exploring adequate ways to do it.

On one side, the metallic compounds already mentioned are being synthesized with one nanoparticle size. And on the other side, the active ingredients associated with nanocarriers (liposomes, miscible suspension, or nanocapsules) need a nanometric system with specific properties that allow them to have a nanotechnology action.

It is essential to mention that compared to the metallic elements, the application of nanotechnology to active ingredients is relatively new. Therefore, the genotoxic evaluation must still be robust enough to generate a risk analysis. Furthermore, especially in Mexico, there is no total selling of these products, and just a few countries worldwide sell them officially. Yet, some active ingredients are still tested to see their efficiency with this technology. Some common ones are pyrethroids, lambda-cyhalothrin, and valinomycin (Demir et al. 2022).

Pyrethroid has been used as nanomicelles in emulsions, and pyrethrin extract obtained from *Chrysanthemum cerium* has been encapsulated in lipid nanocarriers and tested in hematocytes of *Lithobates catesbeianus* tadpoles. Tail length was analyzed by comet assay, finding significant results. Also, endocytosis was observed by transmission microscopy. Results showed that the extract and the capsule alone induced damage and abnormalities in the erythrocytes, opposite to when the encapsulation happened, where the damage decreased (Oliveira et al. 2019).

In another study made with pyrethrin as a nanoemulsion compared with its commercial presentation, its effect was tested in human peripheral blood lymphocytes using the micronucleus test; the results indicated that the commercial presentation was more harmful than the nanoparticulate in a dose-dependent manner (10–100 mg/mL) and that the toxicity of the compound decreased once encapsulated. However, it induced minor genotoxic damage (Sundaramoorthy et al. 2016).

Finally, the last presentation exposed in this paper is nanoencapsulation.

The best advantage of the nanocarriers is to prevent the degradation of the active ingredients through light and temperature, stabilize the compound and allow it to enter the system more quickly.

Each type of carrier has its advantages; the difference between nanoemulsions versus nanoencapsulated is that the former often depend on the suspension in which they are found to maintain their integrity and shape, while nanoencapsulates do not need to remain in suspension since once the medium has evaporated, it can persist in the environment for days.

These encapsulate are the most promising nanotechnological application in pesticides; however, they are still under development. Although technology already implements it in the market, it still needs to stand out in public.

Some examples are Syngenta’s Zeon technology, which consists of 2–3 µm diameter microcapsules with interlaced polymer walls suspended in water and protected by a UV filter. However, up to 100–200 nm capsules have been found (Meredith et al. 2015).

Studies carried out with these nanocapsules are still in process. Still, it has been seen that in the development of zebrafish, lambda-cyhalothrin nanocapsules increased toxicity and decreased the individual’s growth and development rate, leading to teratogenic problems (Meredith et al. 2015).

It has been shown that lambda-cyhalothrin can cause the alteration of the integrity and fluidity in the cell membrane by oxidative stress in rat erythrocytes; in the same way, it produced micronuclei and cytotoxicity (Fetoui et al. 2015).

As soon as the genotoxic potential of lambda-cyhalothrin nanocapsules can be evaluated, the hypothesis that nanotechnology applied to the active ingredient is more harmful than their commercial non-nanometric counterparts can be demonstrated or rejected. In this matter, Paz-Trejo et al. (2022) made the first study to fractionate a commercially encapsulated pesticide by size and compare the genotoxic effect of the unfractionated presentation, the microfraction and the nanofraction on human lymphocytes of peripheral blood; finding that even though the unfractionated product induces damage, it is the nanofraction that potentiates the observed genotoxic damage, from the tiniest concentrations. In other words, the nanocapsules can exert their effect more quickly at the same attention in an in vitro model. Most pesticides currently exert their adverse effect on non-target organisms through oxidative stress or metabolizing the active ingredient. However, nanopesticides with carriers can reach organelles such as the nucleus without being metabolized immediately due to their size and composition, which could induce faster damage directly to the DNA.

Also, they agree that this might be the leading cause for their effect on non-target organisms. Likewise, it is more common to find studies on the product’s effectiveness rather than original research on its impact on the environment or human health; this topic is still pioneering in science and risk assessments. Unfortunately, few works exist on nanopesticides and their effect on human health (Meredith et al. 2015; Sundaramoorthy et al. 2016; Paz-Trejo et al. 2022), and they are all in vitro since there are still no populations fully exposed to these products or not publicly declared, so the existing studies are in animal models such as rats and mice. Despite this, there are still patents ready to go on the market since the studies related to its efficacy in the field have been promising (Chaud et al. 2021). However, the same advantages you may have in attacking pests could be disadvantages for human health.

Although the industry of pesticides associated with nanotechnology is still growing, it is essential to work together to generate a broader vision of their effect on human health, the environment, and non-target organisms.

## Conclusions

Generally, most genotoxic analyses show the damage induced by nanopesticides but not its cause or origin. Instead, most are indirectly associated with oxidative stress and DNA damage.

Most of the damage induced by nanoparticles is seen at low concentrations and half the time as their counterparts. Something important to mention is that most of the genotoxic damage observed is minimal or repairable. However, since it is more remarkable, dysregulation of metabolic pathways that cause cytotoxic damage or cell death must be considered.

Likewise, it should be noted that there is still much research in this regard since more is needed to generate a risk assessment of these products.

Innovation in these products continues to increase, and nanotechnology is only one stage in the production of pesticides. Although, agriculture can be beneficial because it can improve the conditions and optimize the crops. At an environmental and health level, they can induce an unpredictable margin of damage. Therefore, in-depth analysis of these in more animal models and with a greater range of tests is necessary to know whether this technology should be implemented in pesticides or if they will represent an emergent solution or an emergent pollutant.

## Data Availability

Data sharing does not apply to this article as no new data were created or analyzed in this study.
